# COVID-19 Vaccine-Related Psychological Stress Among General Public in China

**DOI:** 10.3389/fpsyt.2021.774504

**Published:** 2021-12-07

**Authors:** Yong-Bo Zheng, Jie Sun, Lin Liu, Yi-Miao Zhao, Wei Yan, Kai Yuan, Si-Zhen Su, Zheng-An Lu, Yue-Tong Huang, Lin Liu, Na Zeng, Xi-Mei Zhu, Yi-Miao Gong, Xiao Lin, Shi-Qiu Meng, Samuel Yeung Shan Wong, Mao-Sheng Ran, Jie Shi, Le Shi, Thomas Kosten, Yan-Ping Bao, Lin Lu

**Affiliations:** ^1^NHC Key Laboratory of Mental Health (Peking University), National Clinical Research Center for Mental Disorders (Peking University Sixth Hospital), Peking University Sixth Hospital, Peking University Institute of Mental Health, Beijing, China; ^2^Peking-Tsinghua Centre for Life Sciences and PKU-IDG/McGovern Institute for Brain Research, Peking University, Beijing, China; ^3^Pain Medicine Center, Peking University Third Hospital, Beijing, China; ^4^National Institute on Drug Dependence and Beijing Key Laboratory of Drug Dependence, Peking University, Beijing, China; ^5^School of Public Health, Peking University, Beijing, China; ^6^Beijing Friendship Hospital, Capital Medical University, Beijing, China; ^7^JC School of Public Health and Primary Care, The Chinese University of Hong Kong, Hong Kong, Hong Kong SAR, China; ^8^Department of Social Work and Social Administration, University of Hong Kong, Hong Kong, Hong Kong SAR, China; ^9^Department of Psychiatry, Baylor College of Medicine, Houston, TX, United States; ^10^Department of Pharmacology, Baylor College of Medicine, Houston, TX, United States; ^11^Department of Neuroscience, Baylor College of Medicine, Houston, TX, United States; ^12^Department of Immunology, Baylor College of Medicine, Houston, TX, United States

**Keywords:** COVID-19 vaccine, psychological stress, vaccination, health knowledge, general public, China

## Abstract

**Background:** The COVID-19 pandemic is our generation's greatest global challenge to our public health system. Vaccines are considered one of the most effective tools available for preventing COVID-19 infection and its complications and sequelae. Understanding and addressing the psychological stress related to COVID-19 vaccination may promote acceptance of these vaccines.

**Methods:** We conducted an online survey from January 29 to April 26, 2021 to explore stress levels related to COVID-19 vaccination among the general public in China. Participants were asked to evaluate their psychological stress of considering whether or not to get vaccinated at the beginning period of the COVID-19 mass vaccination, after getting access to the information about the vaccine, as well as after getting vaccinated, using visual analog stress scale. Multiple linear regression analysis was performed to explore factors potentially associated with COVID-19-related psychological stress levels before and after getting vaccinated.

**Results:** A total of 34,041 participants were included in the final analysis. The mean stress score concerning COVID-19 vaccination was 3.90 ± 2.60 among all participants, and significantly decreased over time. In addition, the vaccine-related stress level significantly decreased after accessing information about the COVID-19 vaccine (*N* = 29,396), as well as after getting vaccinated (*N* = 5,103). Multivariable regression analysis showed higher stress levels related to COVID-19 vaccination in participants who were younger, having lower education level, having history of chronic diseases, mistrusting vaccine's efficacy, experience of vaccine allergy events, being affected by the COVID-19 epidemic, and having mental illness symptoms. Moreover, mistrust in vaccine efficacy and experience of vaccine allergy events had a long-term impact on psychological stress levels about COVID-19 vaccination even after getting vaccinated.

**Conclusions:** The current findings profiled the COVID-19 vaccine-related psychological stress among the general public in China. Population-specific management and interventions targeting the stress related to COVID-19 vaccination are needed to help governments and policy makers promote individual's willingness to get vaccinations for public well-being during the COVID-19 pandemic.

## Introduction

The COVID-19 pandemic is our generation's greatest global challenge to our public health system. As of October 15th, 2021, over 239.4 million people were infected and over 4.8 million individuals were dead of COVID-19 worldwide (1). In China, the number of confirmed infectors was 125.2 thousand, and the number of deaths was about 5.7 thousand as of October 17th, 2021 (2). The Chinese government has implemented timely and effective containment measures since the outbreak of the COVID-19 pandemic, thus the pandemic was long term well-controlled since March 2020 (2). Vaccines are considered one of the most effective tools available for preventing COVID-19 infection and its complications and sequelae (3). Since the first human clinical trial of a COVID-19 vaccine commenced on March 3rd, 2020 (4), a total of 296 COVID-19 vaccines have been developed as clinical and pre-clinical candidates by August 20th, 2021 (1). Despite the validated safety and efficacy of several COVID-19 vaccines, public concern about potential adverse events associated with vaccines still exists (5–7), and affects individuals' willingness, hesitance and refusal to get COVID-19 vaccination (8, 9). Reducing psychological stress or concerns about COVID-19 vaccine would foster confidence and acceptance of vaccination (10). Therefore, it is important to understand COVID-19 vaccine-related stress and identify vulnerable populations with a high stress level to achieve vaccination campaigns success.

Information about COVID-19 vaccine was widely publicized by expert professionals, social media, and government (11, 12). Fake news and insufficient information about COVID-19 vaccine were one of the main causes of adverse psychological responses, and sufficient and transparent news may potentially relieve the associated psychological stress and promote the acceptance of vaccination in some countries (12–14). However, it is unclear how public attitudes toward and psychological stress about COVID-19 vaccines will change when faced with the spread of large amounts of conflicting information about the COVID-19 vaccine (15, 16). It is imperative to profile the details of the psychological stress about COVID-19 vaccination and to explore associated risk factors at the early stages of mass vaccination in China, a country with the largest population in the world.

The World Health Organization declared that over 6.49 billion vaccine doses were already administrated worldwide by October 14th, 2021 (1), and the Chinese government officially announced the number had reached 2.23 billion doses by October 16th, 2021 in China (17). With a substantial number of participants getting vaccinated, their psychological status after vaccination should also be monitored. Despite COVID-19 vaccines being safe for most people aged 18 years and older, rare adverse events still occur. Mild side effects, such as arm soreness, mild fever, tiredness, and headaches are reported after vaccination (18, 19). Moreover, the efficacy of vaccines had not been well-validated in general public before mass vaccinations, and the debate on the efficacy continued even among people who got vaccinated (20). Understanding, describing and addressing the change of psychological stress levels after taking the COVID-19 vaccine among the general public may help the government and policy makers to provide comprehensive and accurate information to those who are hesitant or resistant to getting vaccinated, and build up their confidence in the ongoing vaccination campaign. However, to our knowledge, no current studies have investigated the general public's COVID-19 vaccine-related psychological stress after getting vaccinated.

Based on these considerations, this study had three objectives. First, we sought to identify psychological stress levels and risk factors associated with COVID-19 vaccination when considering getting vaccinated among the general population in China. Second, we sought to determine the influence of accessing information about COVID-19 vaccines on the psychological stress level about vaccination in the general populations. Third, we aimed to explore the change in COVID-19 vaccine-related psychological stress before and after vaccination, as well as to distinguish vulnerable individuals for continued COVID-19 vaccine-related psychological stress after getting vaccinated.

## Methods

### Study Design

This was a cross-sectional, nationwide study conducted via an online survey from January 29 to April 26, 2021, a period when mass vaccination was conducted in China. A self-report questionnaire was designed to investigate COVID-19 vaccine-related psychological stress level among the general public in China, and delivered through Joybuy (http://www.jd.com/), as detailed elsewhere (21, 22). Joybuy platform provides online health products and services with 0.50 billion active users in March, 2021 in China. The study followed the American Association for Public Opinion Research (AAPOR) reporting guidelines and the Strengthening the Reporting of Observational Studies in Epidemiology (STROBE) guidelines. It was approved by the ethics committee of Peking University Sixth Hospital (Institute of Mental Health). Written informed consent was received online before the respondents began the questionnaire.

### Participants

The respondents were all registered members of Joybuy. A total of 74,588 individuals clicked on the survey link, and 34,291 respondents provided informed consent and submitted the questionnaires. Among 34,291 respondents, 4,203 of them provided repeated surveys, and merely the former one was reserved. Two hundred and fifty respondents who were younger than 18 years, were also excluded because obtaining online informed consent from their parents may be not realistic. Finally, a total of 34,041 respondents were included, with the response rate of 46.0% and the effective rate of 99.3%.

### Outcome Measures

The primary outcomes were psychological stress scores, assessed using a visual analog stress scale (23). The stress score ranged from 0 to 10 points, in which 0 represented no stress level and 10 indicated highest level of stress. All participants were asked to evaluate their psychological stress of considering whether or not to get vaccinated at the beginning period of the COVID-19 mass vaccination. Participants who proactively accessed information about the COVID-19 vaccine were asked to evaluate their psychological stress levels after getting access to the information about the vaccine. Moreover, the psychological stress levels of COVID-19 vaccine after getting vaccinated were evaluated among the vaccinated participants.

Additionally, participants were asked to report their sources of stress of considering whether or not to get vaccinated, with the following multiple-choices (16, 24, 25): adverse effects after vaccination of themselves or their families; information about severe adverse effects caused by the vaccine; coverage of vaccine safety incidents reported by the media; misinformation about vaccine-related research reported by the media. The vaccinated populations were also asked to report their sources of stress after getting vaccinated, with the following multiple-choices (24, 25): adverse effects after vaccination of themselves or their families; the efficacy of the vaccine; the safety and quality of the vaccine.

### Covariates

The covariates could be briefly categorized into the following five parts: (1) demographic characteristics and medical conditions, including gender, age, living area (urban vs. rural), education attainment, marital status, and monthly family income, history of chronic diseases, history of mental disorders, and family history of mental disorders; (2) experiences related to the COVID-19 epidemic, including suspect or confirmed infection, infection status of family members or friends, participation in frontline work, job loss due to the COVID-19 epidemic, risk in epidemic regions, experience of quarantine, self-evaluated risk of getting infected, as well as attitudes toward the epidemic in China; (3) information related to the COVID-19 vaccine, including trust in its efficacy after getting vaccinated, experience of being actively involved in getting flu vaccinations, family members experience of being actively involved in getting flu vaccinations, and history of allergic events from previous vaccinations; (4) current mental status: anxiety, depression, insomnia, and posttraumatic stress disorder (PTSD) symptoms; (5) investigation period. According to previous literature (21, 26), cutoff scores of 5 for the Generalized Anxiety Disorder−7 scale, 5 for the Patient Health Questionnaire−9, 8 for the Insomnia Severity Index, and 33 for the Posttraumatic Stress Disorder Checklist for DSM-5 were adopted to detect symptoms of anxiety, depression, insomnia, and PTSD.

### Statistical Analysis

Descriptive statistics were used to present demographic data as well as the sources of psychological stress associated with COVID-19 vaccination. Among all participants, one-way analysis of variance (ANOVA) was used to compare differences of the psychological stress levels among the 3 time groups (Jan. 29–Feb. 28 vs. Mar. 1–Mar. 30 vs. Apr.1–Apr.26). For vaccinated participants, the two-way repeated measures ANOVA with one between-subjects factor (3 time groups: Jan. 29–Feb. 28 vs. Mar. 1–Mar. 30 vs. Apr.1–Apr.26) and one within-subject factor (before vaccination vs. after vaccination) was used to test the differences of psychological stress levels before and after getting vaccinated COVID-19 vaccine at 3 time period. Similarly, repeated measures ANOVA was used to test the differences in psychological stress levels before and after accessing information at 3 time period. Bonferroni *post hoc* analysis was further conducted when the interaction was statistically significant, and *p* values were adjusted using Bonferroni correction with the level of significance of *p* < 0.05 for the comparison.

The mean scores and standard deviation of psychological stress levels associated with COVID-19 vaccination before and after getting vaccinated were calculated and presented in different populations. Analysis of variance and independent *t*-tests were used to compare the psychological stress levels of COVID-19 vaccination before and after getting vaccinated among stratified populations. To explore factors potentially associated with COVID-19-related psychological stress levels before and after getting vaccinated, multiple linear regression analysis was performed, and β values and 95% CIs are presented. No statistical method to handing missing data was used in this analysis because of the limited missing data. Respondents with missing data were furtherly excluded in the multiple linear regression analysis. All of the variables that were statistically significant in the unadjusted model were entered into the multivariable models that explored risk factors associated with vaccine-related stress before and after getting vaccinated. Multicollinearity between the independent variables was checked by calculating the variance inflation factor (VIF), and VIF > 5 indicated multicollinearity (27). Separate models excluding highly correlated covariates were performed if included independent variables were multicollinear. The level of significance was *p* < 0.05. All of the statistical analyses were performed using SPSS statistical software version 22 (IBM Corp).

## Results

### Demographic Characteristics

34,041 participants from 34 provinces in China were included in the final analysis, of whom 40.4, 51.1, and 8.5% responded to the survey during Jan. 29–Feb. 28, Mar. 1–Mar. 30, and Apr. 1–Apr. 26, respectively. Of the total sample, most of the participants were female (53.8%), aged between 18 and 39 (60.9%), lived in an urban area (79.1%), had a college degree or higher (79.2%), and were married (77.5%). 29,396 participants (86.4%) actively accessed information about the COVID-19 vaccine. 78.4 and 17.3% of the participants highly and moderately trusted the efficacy of the COVID-19 vaccine and agreed that vaccination was an effective measure for COVID-19 prevention, 4.3% did not trust the efficacy of the COVID-19 vaccine. 5,103 (15.0%) participants had been vaccinated against COVID-19, and about one third of the participants (11,515) had obtained a flu vaccination. 4,050 participants (11.9%) reported their experience of vaccine allergy events. In addition, 21.1, 23.1, 27.5, and 29.5% of participants reported symptoms of anxiety, depression, insomnia, and PTSD, respectively. The demographic characteristics, medical conditions, COVID-19 epidemic-related information, vaccine-related information, and mental status of the total samples are presented in Table 1, and of the vaccinated participants in Supplementary Table 1.

**Table 1:** Characteristics and population-stratified COVID-19 vaccine-related psychological stress level when considering vaccine uptake among all participants.

**Factors**	**Total, no. (%)**	**Stress score (***SD***)**	***P***
**Overall**	34,041 (100.0)	3.90 (2.60)	
**Gender**			0.842
Female	18,309 (53.8)	3.90 (2.55)	
Male	15,732 (46.2)	3.89 (2.66)	
**Age**			<0.001
18–39 years	20,727 (60.9)	3.96 (2.61)	
40–59 years	12,713 (37.3)	3.82 (2.57)	
≥60 years	601 (1.8)	3.50 (2.67)	
**Living area**			0.992
Urban	26,942 (79.1)	3.90 (2.59)	
Rural	7,099 (20.9)	3.90 (2.63)	
**Level of education**			<0.001
Less than college	7,084 (20.8)	4.04 (2.67)	
College degree or higher	26,957 (79.2)	3.86 (2.58)	
**Marital status**			0.951
Married	26,392 (77.5)	3.90 (2.59)	
Unmarried	7,649 (22.5)	3.90 (2.64)	
**Monthly family income**, ¥^a^			<0.001
0–4,999	8,438 (24.8)	4.09 (2.68)	
5,000–11,999	15,961 (46.9)	3.91 (2.57)	
≥12,000	9,642 (28.3)	3.71 (2.58)	
**History of chronic diseases**			<0.001
No or unknown	30,938 (90.9)	3.87 (2.60)	
Yes	3,103 (9.1)	4.14 (2.62)	
**History of mental disorders**			<0.001
No or unknown	33,873 (99.5)	3.89 (2.60)	
Yes	168 (0.5)	4.90 (2.76)	
**Family history of mental disorders**			<0.001
No or unknown	33,614 (98.7)	3.89 (2.60)	
Yes	427 (1.3)	4.78 (2.73)	
**Have you been infected with COVID-19?**			<0.001
No	33,937 (99.7)	3.89 (2.60)	
Suspect or confirmed infected	104 (0.3)	5.13 (2.72)	
**Have any of your family members or friends been infected with COVID-19?**			<0.001
No	33,618 (98.8)	3.89 (2.60)	
Yes	423 (1.2)	4.73 (2.68)	
**Have you been a frontline worker since august 2020?**			0.161
No	28,261 (83.0)	3.91 (2.57)	
Yes	5,780 (17.0)	3.85 (2.75)	
**Has the epidemic led to your job loss since august 2020?**			<0.001
No	31,253 (91.8)	3.84 (2.59)	
Yes	2,788 (8.2)	4.53 (2.68)	
**Risk in epidemic regions**			<0.001
Low	33,346 (98.0)	3.87 (2.59)	
Middle/High	695 (2.0)	5.20 (2.60)	
**Have you ever experienced quarantine since august 2020?**			<0.001
No	30,160 (88.6)	3.85 (2.59)	
Yes	3,881 (11.4)	4.25 (2.68)	
**Evaluate your risk of getting infected in the future**			<0.001
Low	30,602 (89.9)	3.78 (2.59)	
Middle/High	3,439 (10.1)	4.90 (2.52)	
**Attitudes toward the epidemic in China ^b^**			<0.001
Positive	14,373 (42.2)	3.63 (2.64)	
Neutral	18,117 (53.2)	4.06 (2.52)	
Negative	1,551 (4.6)	4.45 (2.89)	
**Do you trust in efficacy of COVID-19 vaccine?**			<0.001
No	1,472 (4.3)	5.15 (2.86)	
Moderate	5,887 (17.3)	4.64 (2.47)	
Highly	26,682 (78.4)	3.66 (2.56)	
**Have you ever been actively involved in getting flu vaccination?**			0.165
No	22,526 (66.2)	3.91 (2.75)	
Yes	11,515 (33.8)	3.87 (2.57)	
**Have your family members ever been actively involved in getting flu vaccination?**			<0.001
No	18,551 (54.5)	3.96 (2.58)	
Yes	15,490 (45.5)	3.82 (2.63)	
**Have you ever had any allergy events from previous vaccinations?**			<0.001
No	29,991 (88.1)	3.74 (2.55)	
Yes	4,050 (11.9)	5.06 (2.69)	
**Anxiety symptoms**			<0.001
No	26,848 (78.9)	3.50 (2.52)	
Yes	7,193 (21.1)	5.39 (2.35)	
**Depressive symptoms**			<0.001
No	26,178 (76.9)	3.49 (2.52)	
Yes	7,863 (23.1)	5.25 (2.40)	
**Insomnia symptoms**			<0.001
No	24,693 (72.5)	3.51 (2.55)	
Yes	9,348 (27.5)	4.93 (2.43)	
**PTSD symptoms**			<0.001
No	24,009 (70.5)	3.40 (2.53)	
Yes	10,032 (29.5)	5.10 (2.37)	
**Investigation period**			<0.001
January 29, 2021–February 28, 2021	13,739 (40.4)	4.17 (2.58)	
March 1, 2021–March 31, 2021	17,396 (51.1)	3.76 (2.60)	
April 1, 2021–April 26, 2021	2,906 (8.5)	3.45 (2.57)	

*COVID-19, coronavirus disease 2019; PTSD, posttraumatic stress disorder; SD, standard derivation*.

a *1 ¥ = USD$0.14*.

b *Participants who thought the COVID-19 epidemic would end within 1 year, 1–10 years, and over 10 years or long lasting were defined as positive, neutral, and negative attitudes toward, respectively*.

### The Sources of COVID-19 Vaccine-Related Psychological Stress

**Figure 1:**
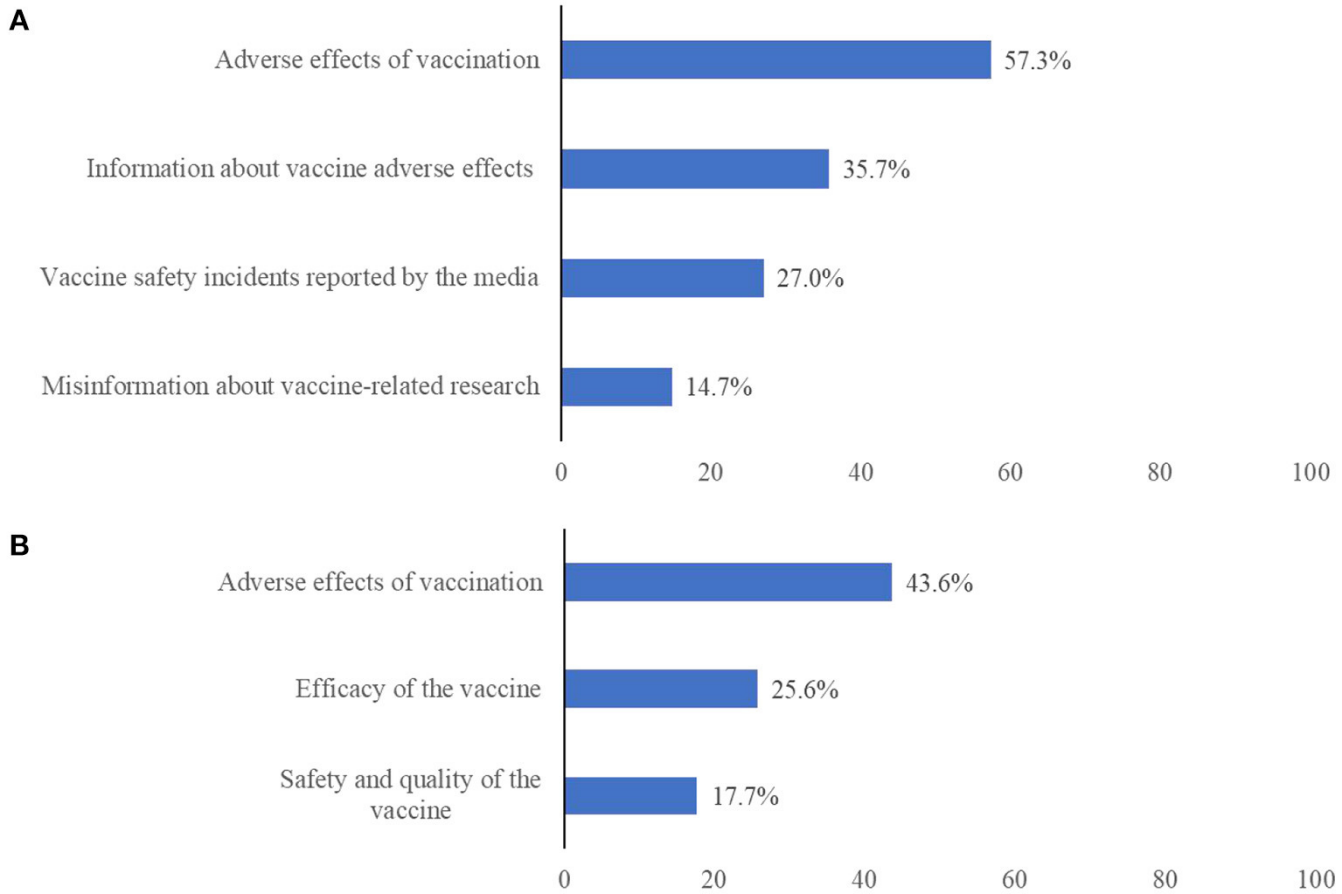
The sources of psychological stress associated with COVID-19 vaccine uptake **(A)** at the beginning period of the COVID-19 mass vaccination (*N* = 34,041), and **(B)** after vaccination (*N* = 5,103).

81.3% of all participants experienced any psychological stress about vaccination. The sources of this psychological stress about the COVID-19 vaccine were ranked as follows (Figure 1A): 57.3% were concerned about the adverse effects after vaccination of themselves or their families; 35.7% were concerned by the news of severe adverse effects associated with the vaccine; 27.0% were concerned by vaccine safety incidents reported in the media; and 14.7% of participants were concerned by some misinformation from vaccine-related research. After getting the COVID-19 vaccine, 58.6% of participants had psychological stress and the reasons for psychological stress about the COVID-19 vaccination were ranked as follows (Figure 1B): 43.6% of participants were concerned about the adverse effects in themselves or their families after vaccination; 25.6% of participants worried about the efficacy of vaccine; and 17.7% of participants concerned the safety and quality of vaccine.

### The COVID-19 Vaccine-Related Psychological Stress Levels

**Figure 2:**
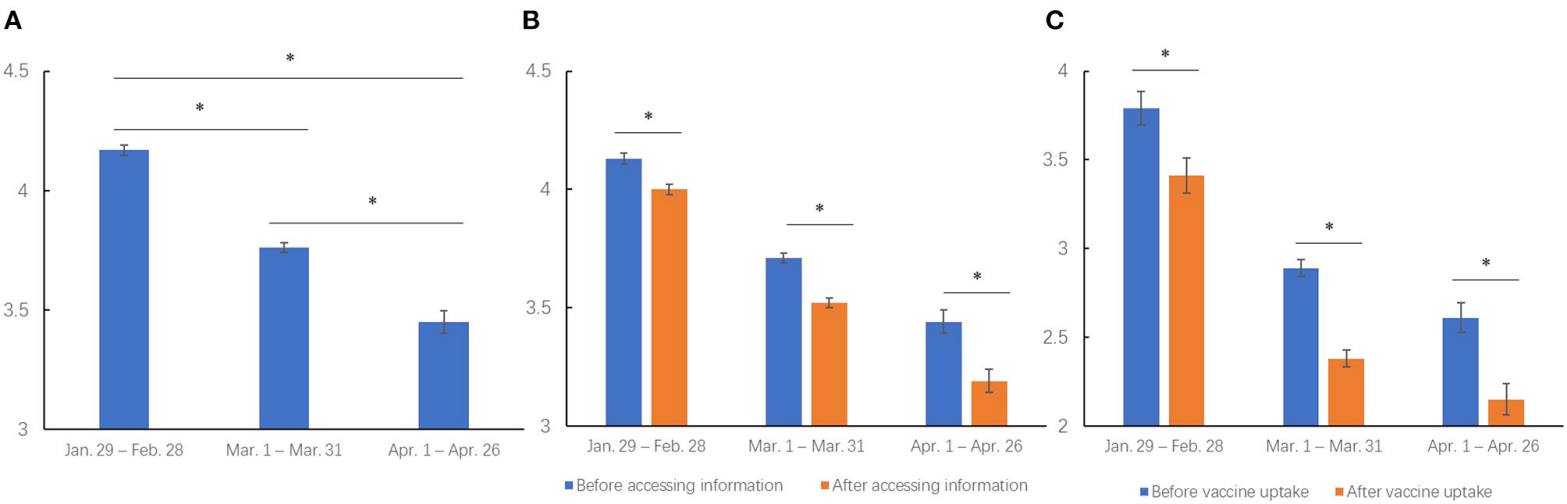
COVID-19 vaccine-related psychological stress levels **(A)** at the beginning period of the COVID-19 mass vaccination (*N* = 34,041), **(B)** before and after accessing information about vaccination (*N* = 29,396), **(C)** before and after getting vaccinated (*N* = 5,103) from Jan. 29 to Apr. 26, 2021. *indicated statistically significant with *p* < 0.05.

The mean stress score concerning COVID-19 vaccination was 3.90 ± 2.60 among all participants. The stress levels about vaccination were significantly decreased from Jan. 29 to Apr. 26 (Jan. 29–Feb. 28: 4.17 ± 2.58, Mar. 1–Mar. 30: 3.76 ± 2.60, Apr. 1–Apr. 26: 3.45 ± 2.57; [*F*_(2,34038)_ = 142.90, *p* < 0.001, Figure 2A]), and *post hoc* analysis found that comparisons of vaccine uptake stress levels between any 2 months were significantly different, with all *p* < 0.001 by using Bonferroni's correction.

The 29,396 participants who actively accessed information about COVID-19 vaccines, significantly decreased their psychological stress levels after accessing associated information when compared to stress levels before the access, and the levels also decreased over time from Jan. 29 to Apr. 26 (information accessing: [*F*_(1,29393)_ = 295.39, *p* < 0.001]; time: [*F*_(2,29393)_ = 162.32, *p* < 0.001]; interaction of information accessing and time: [*F*_(2,29393)_ = 7.11, *p* = 0.001; Figure 2B]). *Post hoc* analysis found that the stress level was significantly decreased after accessing the information when compared to that before at all 3 months (before vs. after: from 4.13 ± 2.55 to 4.00 ± 2.48 during Jan. 29–Feb. 28, from 3.71 ± 2.58 to 3.52 ± 2.50 during Mar. 1–Mar. 30, from 3.44 ± 2.54 to 3.19 ± 2.52 during Apr.1–Apr. 26, all *p* < 0.001 by Bonferroni's correction), and was decreased during the 3 months from Jan. 29 to Apr. 26.

In addition, the 5,103 vaccinated participants had significantly decreased psychological stress levels about COVID-19 vaccination after getting vaccinated than before vaccination at all 3 months (vaccine uptake: [*F*_(1,5100)_ = 231.29, *p* < 0.001]; time: [*F*_(2,5100)_ = 65.22, *p* < 0.001]; interaction of vaccine uptake and time: [*F*_(2,5100)_ = 2.06, *p* = 0.127]; from 3.79 ± 2.91 to 3.41 ± 3.07 during Jan. 29–Feb. 28, from 2.89 ± 2.66 to 2.38 ± 2.70 during Mar. 1–Mar. 30, from 2.61 ± 2.56 to 2.15 ± 2.61 during Apr. 1–Apr. 26; Figure 2C).

### Associated Factors With the COVID-19-Related Psychological Stress Level

Table 2 shows the associated factors with the level of COVID-19 vaccine-related psychological stress when considering getting vaccinated at the beginning period of the COVID-19 mass vaccination among general public. Multiple linear regression analysis showed that older adults (β = −0.38, *p* < 0.001) displayed a lower level of COVID-19-related psychological stress. Participants with a history of chronic diseases (β = 0.10, *p* = 0.031) and low education level (β = −0.08, *p* = 0.019) had significantly higher psychological stress levels. Several epidemic-related factors were associated with psychological stress levels about COVID-19 vaccination, including experience of job loss due to the COVID-19 epidemic (β = 0.24, *p* < 0.001), quarantine experience (β = 0.11, *p* = 0.008), and self-evaluated high risk of COVID-19 infection (β = 0.50, *p* < 0.001). In addition, individuals with neutral or negative attitudes toward the epidemic in China had increased psychological stress levels (neutral: β = 0.26, *p* < 0.001; negative: β = 0.38, *p* < 0.001) compared to those with positive attitudes toward the epidemic in China.

**Table 2:** Multivariable linear regression of factors associated with psychological stress levels of COVID-19 vaccination at the beginning period of the COVID-19 mass vaccination among general public.

	**β (95% CI)**	***P***	**VIF**
40–59 years (ref: 18–39 years)	0.001 (−0.054, 0.056)	0.974	1.08
≥60 years (ref: 18–39 years)	**−0.378 (−0.575, −0.180)**	**0.001**	1.05
College degree or higher (ref: less than college)	**−0.079 (−0.145, −0.013)**	**0.019**	1.12
5,000–11,999 monthly family income, ¥^a^ (ref: 0–4,999 monthly family income)	0.007 (−0.058, 0.072)	0.833	1.62
≥12,000 monthly family income, ¥^a^ (ref: 0–4,999 monthly family income)	−0.058 (−0.132, 0.016)	0.123	1.71
History of chronic diseases (ref: no)	**0.100 (0.009, 0.190)**	**0.031**	1.06
History of mental disorders (ref: no)	−0.054 (−0.425, 0.317)	0.776	1.05
Family history of mental disorders (ref: no)	0.093 (−0.140, 0.327)	0.433	1.05
Suspect or confirmed infected with COVID-19 (ref: no)	−0.267 (−0.744, 0.211)	0.274	1.07
Family members or friends infected with COVID-19 (ref: no)	0.202 (−0.035, 0.438)	0.095	1.06
Job loss due to the COVID-19 epidemic (ref: no)	**0.237 (0.142, 0.332)**	**<0.001**	1.04
Middle/high- risk in epidemic regions (ref: low-risk)	0.166 (−0.020, 0.351)	0.080	1.07
Quarantine experience (ref: no)	**0.111 (0.029, 0.193)**	**0.008**	1.05
Self-evaluated middle/high risk of getting infected (ref: low risk)	**0.504 (0.417, 0.591)**	**<0.001**	1.07
Neutral attitudes toward the epidemic in China (ref: positive)	**0.256 (0.203, 0.309)**	**<0.001**	1.09
Negative attitudes toward the epidemic in China (ref: positive)	**0.376 (0.250, 0.503)**	**<0.001**	1.08
Moderate trust in efficacy of the COVID-19 vaccine (ref: distrust)	**−0.259 (−0.396, −0.121)**	**<0.001**	4.17
Highly trust in efficacy of the COVID-19 vaccine (ref: distrust)	**−0.978 (−1.105, −0.851)**	**<0.001**	4.25
Family members experience of actively involved in getting flu vaccination (ref: no)	**−0.072 (−0.123, −0.020)**	**0.006**	1.02
Experience of vaccine allergy events (ref: no)	**0.707 (0.626, 0.788)**	**<0.001**	1.07
Anxiety symptoms (ref: no)	**0.713 (0.605, 0.820)**	**<0.001**	2.97
Depressive symptoms (ref: no)	**0.203 (0.096, 0.310)**	**<0.001**	3.16
Insomnia symptoms (ref: no)	**0.315 (0.243, 0.387)**	**<0.001**	1.61
PTSD symptoms (ref: no)	**0.772 (0.694, 0.850)**	**<0.001**	1.95
Investigation period	**−0.209 (−0.250, −0.167)**	**<0.001**	1.03

*COVID-19, coronavirus disease 2019; PTSD, posttraumatic stress disorder; VIF, variance inflation factor*.

a *1 ¥ = USD$0.14*.

*Bold values indicated statistically significant with p < 0.05*.

Regarding the information about the COVID-19 vaccine, trust in the COVID-19 vaccine's efficacy was associated with an individual's psychological stress level about vaccination. Specifically, individuals with moderate or high trust in the efficacy of the COVID-19 vaccine in the prevention of infection displayed a lower psychological stress level (moderate: β = −0.26, *p* < 0.001; highly: β = −0.98, *p* < 0.001) compared to the participants who mistrusted the COVID-19 vaccine. In addition, participants with experiences of family members who were actively involved in flu vaccination reported lower psychological stress levels, compared with participants without these experiences (β = −0.07, *p* = 0.006). Participants with experience of vaccine allergy events had a significantly elevated psychological stress level (β = 0.71, *p* < 0.001). Participants with any mental symptoms (anxiety: β = 0.71, *p* < 0.001; depression: β = 0.20, *p* < 0.001; insomnia: β = 0.32, *p* < 0.001; PTSD: β = 0.77, *p* < 0.001) had significantly higher psychological stress levels about COVID-19 vaccination. Moreover, psychological stress level about vaccination decreased over time during the investigation period (β = −0.21, *p* < 0.001).

The factors associated with psychological stress levels about COVID-19-vaccine after vaccination among the vaccinated participants are presented in Table 3. VIF of all factors suggested no significant collinearity. Participants with high trust in the efficacy of the COVID-19 vaccine showed significantly lower psychological stress levels (β = −0.43, *p* = 0.007). Significantly higher psychological stress levels occurred among those with high psychological stress levels at the beginning period of vaccination (β = 0.73, *p* < 0.001), experiences of vaccine allergy events (β = 0.55, *p* <0.001), anxiety symptoms (β = 0.51, *p* <0.001), and PTSD symptoms (β = 0.35, *p* <0.001).

**Table 3:** Multivariable linear regression of factors associated with COVID-19 vaccine-related psychological stress after vaccination among the vaccinated participants.

	**β (95% CI)**	***P***	**VIF**
COVID-19 vaccine related stress level before getting vaccinated	**0.725 (0.706, 0.744)**	**<0.001**	1.24
40–59 years (ref: 18–39 years)	−0.014 (−0.111, 0.083)	0.777	1.09
≥60 years (ref: 18–39 years)	−0.178 (−0.584, 0.229)	0.391	1.04
College degree or higher (ref: less than college)	−0.099 (−0.231, 0.033)	0.143	1.16
5,000–11,999 monthly family income, ¥^a^ (ref: 0–4,999 monthly family income)	−0.067 (−0.190, 0.056)	0.285	1.78
≥12,000 monthly family income, ¥^a^ (ref: 0–4,999 monthly family income)	−0.126 (−0.262, 0.009)	0.068	1.87
History of mental disorders (ref: no)	0.251 (−0.393, 0.896)	0.445	1.07
Family history of mental disorders (ref: no)	−0.059 (−0.476, 0.358)	0.781	1.08
Suspect or confirmed infected with COVID-19 (ref: no)	0.181 (−0.536, 0.898)	0.620	1.09
Family members or friends infected with COVID-19 (ref: no)	0.290 (−0.062, 0.642)	0.106	1.08
Job loss due to COVID-19 epidemic (ref: no)	0.129 (−0.071, 0.329)	0.206	1.07
Middle/high- risk in epidemic regions (ref: low-risk)	0 (−0.383, 0.382)	0.998	1.08
Quarantine experience (ref: no)	0.108 (−0.030, 0.246)	0.124	1.06
Self-evaluated middle/high risk of getting infected (ref: low risk)	0.079 (−0.079, 0.237)	0.325	1.06
Neutral attitudes toward the epidemic in China (ref: positive)	−0.022 (−0.117, 0.074)	0.657	1.07
Negative attitudes toward the epidemic in China (ref: positive)	0.019 (−0.234, 0.272)	0.883	1.06
Moderate trust in efficacy of the COVID-19 vaccine (ref: distrust)	−0.135 (−0.489, 0.220)	0.457	3.21
Highly trust in efficacy of the COVID-19 vaccine (ref: distrust)	**−0.417 (−0.722, −0.112)**	**0.007**	3.30
Experience of actively involved in getting flu vaccination (ref: no)	0.055 (−0.039, 0.148)	0.250	1.02
Experience of vaccine allergy events (ref: no)	**0.551 (0.385, 0.717)**	**<0.001**	1.18
Anxiety symptoms (ref: no)	**0.514 (0.313, 0.715)**	**<0.001**	2.77
Depressive symptoms (ref: no)	0.060 (−0.135, 0.255)	0.544	2.88
Insomnia symptoms (ref: no)	0.074 (−0.057, 0.204)	0.267	1.49
PTSD symptoms (ref: no)	**0.350 (0.210, 0.489)**	**<0.001**	1.80
Investigation period	**−0.084 (−0.162, −0.007)**	**0.033**	1.05

*COVID-19, coronavirus disease 2019; PTSD, posttraumatic stress disorder; VIF, variance inflation factor*.

a *1 ¥ = USD$0.14*.

*Bold values indicated statistically significant with p <0.05*.

## Discussion

The present study investigated COVID-19 vaccine-related psychological stress levels among the general population in China based on a nationwide, large-sample survey. The psychological stress level of COVID-19 vaccination significantly decreased over time, after accessing information about the COVID-19 vaccine, as well as after getting vaccinated. Several risk factors contributing to the psychological stress level of COVID-19 vaccination when considering getting vaccinated were identified, including younger age, lower education level, history of chronic diseases, mistrust in vaccine efficacy, experience of vaccine allergy events, being affected by the COVID-19 epidemic, and having mental illness symptoms. Moreover, mistrust in vaccine efficacy and experience of vaccine allergy events had a long-term impact on psychological stress levels about COVID-19 vaccination even after getting vaccinated. These findings provide a comprehensive profile of COVID-19 vaccine-related psychological stress levels before and after getting vaccinated and may contribute to promoting the willingness to be vaccinated and improve the general population's well-being during the COVID-19 pandemic.

The psychological stress level of COVID-19 vaccination may lead to the hesitation and rejection of vaccination (28). Due to the COVID-19 experience and ignorance about vaccines, the psychological stress about COVID-19 vaccination was common at the beginning of COVID-19 mass vaccinations. Despite the widely validated efficacy of the COVID-19 vaccine, some individuals still mistrusted the efficacy of the COVID-19 vaccine (5–7, 29). Participants who held negative attitudes toward the efficacy of the COVID-19 vaccine had significantly higher psychological stress levels about vaccination. However, previous research has suggested that accessing information about COVID-19 vaccine generally had both good and bad effects, since fake news increased psychological stress levels, while accurate information reduced individuals' psychological stress levels (12, 15, 16). Promoting the efficacy of the COVID-19 vaccine built up the confidence and reduced the psychological stress of vaccination (10). The results of this survey showed that the psychological stress level decreased after vaccination, which indicates that the observed safety of vaccination in real life may relieve the misinformation and associated psychological stress level. Therefore, combating misinformation and disseminating accurate information about the COVID-19 vaccine will reduce psychological stress levels about COVID-19 vaccination in the general population and promote vaccination programs.

Consistent with early findings (12), the results of this study showed that the fear of adverse effects was another strong source of increased psychological stress about the COVID-19 vaccination even after getting vaccinated. Participants with experiences of vaccine allergy events had a significantly elevated psychological stress level when considering getting the COVID-19 vaccine (18, 19). Severe adverse effects generally occurred immediately or over a short period after getting vaccinated (18, 19), and the psychological stress level of COVID-19 vaccination among the vaccinated participants with no adverse effects decreased after vaccination. However, some participants still experienced psychological stress even after getting COVID-19 vaccination due to the participants mistrusting the efficacy of the vaccination and experiencing vaccine allergy events. The findings further imply the importance of guarantee the efficacy and safety of the vaccines (10, 12). For participants with consistent psychological stress about the COVID-19 vaccine, specific strategies and policies should be made to help relieve their psychological stress even after getting vaccinated.

Moreover, we found that family members' experiences of involvement in flu vaccination had a positive effect on individual's psychological stress about COVID-19 vaccination. We proposed that families, as a unit, to get vaccinated may be helpful to relieve other family members' psychological stress about the COVID-19 vaccination. In addition, acceptance of the vaccine among family members, especially parents, would have a positive effect on their children's vaccination in the future (30).

Except for information about the vaccine, the pandemic itself may have long-term impacts on individuals' psychological status (21, 31). In this study, epidemic-related factors, including job loss due to the COVID-19 epidemic, experience of quarantine, self-evaluated high risk of getting infected, and negative attitudes toward the epidemic in China were associated with elevated psychological stress levels when considering the COVID-19 vaccination. The COVID-19 pandemic may have both negative (e.g., increased risk of vaccine-preventable diseases outbreaks) and positive effects (e.g., need for a coronavirus vaccine may increase people's appreciation for vaccines in general) on individual willingness for vaccination; however, it still unclear which effect is dominant (32). This study indicates that mental health status during the COVID-19 pandemic will impact psychological stress levels about COVID-19 vaccination in the general population. Individuals with health issues (e.g., chronic physical or mental illness) were at greater risk of being infected with COVID-19, thus these populations deserve to be in the priority groups for vaccination (33). Given the urgent need and psychological stress of vaccination among the general population, it is crucial for government and policy makers to facilitate COVID-19 vaccination and reduce the relevant psychological stress.

This study showed that some demographic factors and history of chronic diseases may also influence the psychological stress of vaccination. Older adults were regarded as the critical group for determining the success of this vaccine campaign (34). In this study, older adults had decreased COVID-19 vaccine-related psychological stress levels. However, the old adults were generally found to be less willing to get vaccinated (35). We suspect that the discrepancy of acceptance and psychological stress about COVID-19 vaccination could be related to the co-existence of better stress resilience and vaccine apathy among older adults (13). Similarly, individuals with low education levels had greater psychological stress levels about vaccination, which could be explained by poor awareness and health literacy, lower trust and interaction with healthcare professionals, and cost-based concerns among them (36). Generally, comorbidity did not affect individuals' acceptance of vaccine uptake (8), but may increase unrelated psychological stress about their comorbid illnesses. Thus, more strategies and interventions should be developed to relieve psychological stress about vaccination in those with history of chronic disease.

The current findings have potential implications for vaccine rollout policies in China and other countries. First, to build public confidence in vaccine programs and relive vaccine related stress, the government officials should guarantee the safety and effectiveness of vaccines (25). Second, as the main avenues of delivering COVID-19 vaccine-related information, the social media should disseminate accurate and proper information about the COVID-19 vaccine (11). Third, the government and health authorities should keep more supervision on specific targeted populations, even after getting vaccinated. Last but not least, more researches on vaccine-related psychological problems were proposed.

The strengths of this study include its extensive geographic coverage across China, and large sample size. Participants with different characteristics were recruited from all 34 province-level regions in China. In addition, to the best of our knowledge, this is the first study that systematically investigated the COVID-19 vaccine-related psychological stress level. However, our study has several limitations. First, this was an online survey via Joybuy platform, and we used a convenience sampling method. Although this study had extensive geographic coverage across China and a large sample size, most respondents were young, highly educated, living urban areas, with no history of mental disorders, non-infectors, as well as actively involved in accessing information about the vaccine; thus, the representativeness of the sample might be limited, and self-selection bias would exist. Second, we assessed the psychological stress levels using self-reported visual analog scales, rather than well-constructed tools. Third, this was a cross-sectional study. Therefore, associations between psychological stress levels when considering vaccine uptake and risk factors cannot necessarily be considered causal relationships. Fourth, the recall bias cannot be avoided, as the stress vaccine-related stress at different occasions were recalled and self-reported by individuals at one-time point investigation.

## Conclusions

The current findings profiled the COVID-19 vaccine-related psychological stress among the general public in China. This information can provide help for policy making, recognition of vulnerable populations, and framework design for population-specific management to reduce the COVID-19 vaccine-related psychological stress levels and promote the acceptance of the vaccine and improve public health well-being during the COVID-19 pandemic.

## Data Availability Statement

The original contributions presented in the study are included in the article/Supplementary Material, further inquiries can be directed to the corresponding authors.

## Ethics Statement

The studies involving human participants were reviewed and approved by Peking University Sixth Hospital. The patients/participants provided their written informed consent to participate in this study. Written informed consent was obtained from the individual(s) for the publication of any potentially identifiable images or data included in this article.

## Author Contributions

Y-BZ, JSu, TK, Y-PB, and LLu designed the protocol. Y-BZ, LL (third author), Y-MZ, Y-TH, and LL (tenth author) were involved in data collection. Y-BZ, Y-MZ, S-ZS, Z-AL, and NZ analyzed the data. Y-BZ, JSu, LL (third author), WY, and KY drafted the manuscript. X-MZ, XL, S-QM, SW, M-SR, JSh, LS, TK, Y-BZ, and LLu revised the manuscript. All the authors have read and approved the final version of the manuscript.

## Funding

This study was supported by grants from the National Key Research and Development Program of China (Nos. 2021YFC0863700 and 2019YFA0706200) and the National Natural Science Foundation of China (Nos. 81761128036, 82171514, 81821092, and 31900805).

## Conflict of Interest

The authors declare that the research was conducted in the absence of any commercial or financial relationships that could be construed as a potential conflict of interest.

## Publisher's Note

All claims expressed in this article are solely those of the authors and do not necessarily represent those of their affiliated organizations, or those of the publisher, the editors and the reviewers. Any product that may be evaluated in this article, or claim that may be made by its manufacturer, is not guaranteed or endorsed by the publisher.
