# Supplementary material for: COVID-19 Vaccine-Related Psychological Stress Among General Public in China
Source: Front Psychiatry. 2021 Dec 7;12:774504. doi: 10.3389/fpsyt.2021.774504 (PMC8689133; doi:10.3389/fpsyt.2021.774504)
Supplement: Supplementary file 1 [file Table_1.DOCX]

**Supplement Table 1. C****haracteristics and population-stratified COVID-19 vaccine-related psychological stress level after vaccination among the vaccinated participants.**

| **Factors** | **Vaccinated participants, No. (%)** | **Stress score (SD)** | **P value** |
| --- | --- | --- | --- |
| **Overall** | 5103 (100.0) | 2.53 (2.79) |  |
| **Gender** |  |  | 0.109 |
| Female | 2398 (47.0) | 2.46 (2.76) |  |
| Male | 2705 (53.0) | 2.59 (2.82) |  |
| **Age** |  |  | < 0.001 |
| 18-39 years | 2826 (55.4) | 2.75 (2.90) |  |
| 40-59 years | 2208 (43.3) | 2.25 (2.61) |  |
| ≥ 60 years | 69 (1.4) | 2.45 (2.95) |  |
| **Living area** |  |  | 0.170 |
| Urban | 4077 (79.9) | 2.50 (2.78) |  |
| Rural | 1026 (20.1) | 2.63 (2.80) |  |
| **Level of education** |  |  | < 0.001 |
| Less than college | 860 (16.9) | 3.03 (3.09) |  |
| College degree or higher | 4243 (83.1) | 2.43 (2.71) |  |
| **Marital status** |  |  | 0.073 |
| Married | 4009 (78.6) | 2.49 (2.75) |  |
| Unmarried | 1094 (21.4) | 2.66 (2.92) |  |
| **Monthly family income, ¥**^a^ |  |  | < 0.001 |
| 0-4999 | 1141 (22.4) | 2.98 (3.00) |  |
| 5000-11999 | 2359 (46.2) | 2.55 (2.79) |  |
| ≥ 12000 | 1603 (31.4) | 2.18 (2.58) |  |
| **History of chronic diseases** |  |  | 0.876 |
| No or unknown | 4689 (91.9) | 2.53 (2.79) |  |
| Yes | 414 (8.1) | 2.55 (2.76) |  |
| **History of mental disorders** |  |  | < 0.001 |
| No or unknown | 5075 (99.5) | 2.52 (2.78) |  |
| Yes | 28 (0.5) | 4.82 (3.38) |  |
| **Family history of mental disorders** |  |  | < 0.001 |
| No or unknown | 5035 (98.7) | 2.50 (2.77) |  |
| Yes | 68 (1.3) | 4.51 (3.40) |  |
| **Have you been infected with COVID-19?** |  |  | < 0.001 |
| No | 5080 (99.5) | 2.52 (2.78) |  |
| Suspect or confirmed infected | 23 (0.5) | 5.04 (3.67) |  |
| **Have any of your family members or friends been infected with COVID-19?** |  |  | < 0.001 |
| No | 28611 (98.9) | 2.50 (2.77) |  |
| Yes | 327 (1.1) | 3.89 (3.23) |  |
| **Have you been a frontline worker since August 2020?** |  |  | 0.718 |
| No | 3313 (64.9) | 2.52 (2.76) |  |
| Yes | 1790 (35.1) | 2.55 (2.84) |  |
| **Has the epidemic led to your job loss since August 2020?** |  |  | < 0.001 |
| No | 4795 (94.0) | 2.44 (2.73) |  |
| Yes | 308 (6.0) | 3.90 (3.34) |  |
| **Risk in epidemic regions** |  |  | < 0.001 |
| Low | 5022 (98.4) | 2.49 (2.77) |  |
| Middle/High | 81 (1.6) | 4.62 (3.21) |  |
| **Have you ever experienced quarantine since August 2020?** |  |  | < 0.001 |
| No | 4405 (86.3) | 2.42 (2.71) |  |
| Yes | 698 (13.7) | 3.20 (3.15) |  |
| **Evaluate your risk of getting infected in the future** |  |  | < 0.001 |
| Low | 4594 (90.0) | 2.42 (2.75) |  |
| Middle/High | 509 (10.0) | 3.49 (2.97) |  |
| **Attitudes towards the epidemic in China** |  |  | < 0.001 |
| Positive | 2324 (45.5) | 2.42 (2.85) |  |
| Neutral | 2593 (50.8) | 2.60 (2.72) |  |
| Negative | 186 (3.6) | 2.87 (2.97) |  |
| **Do you trust in efficacy of COVID-19 vaccine?** |  |  | < 0.001 |
| No | 124 (2.4) | 4.58 (3.44) |  |
| Moderate | 293 (5.7) | 4.17 (2.96) |  |
| Highly | 4686 (91.8) | 2.37 (2.70) |  |
| **Have you ever been actively involved in getting flu vaccination?** |  |  | < 0.001 |
| No | 2760 (54.1) | 2.35 (2.61) |  |
| Yes | 2343 (45.9) | 2.74 (2.97) |  |
| **Have your family members ever been actively involved in getting flu vaccination?** |  |  | 0.781 |
| No | 2252 (44.1) | 2.52 (2.70) |  |
| Yes | 2851 (55.9) | 2.54 (2.86) |  |
| **Have you ever had any allergy events from previous vaccinations?** |  |  | < 0.001 |
| No | 4586 (89.9) | 2.26 (2.61) |  |
| Yes | 517 (10.1) | 4.93 (3.11) |  |
| **Anxiety symptoms** |  |  | < 0.001 |
| No | 4205 (82.4) | 2.04 (2.50) |  |
| Yes | 898 (17.6) | 4.81 (2.92) |  |
| **Depressive symptoms** |  |  | < 0.001 |
| No | 4082 (80.0) | 2.03 (2.51) |  |
| Yes | 1021 (20.0) | 4.51 (2.97) |  |
| **Insomnia symptoms** |  |  | < 0.001 |
| No | 3843 (75.3) | 2.10 (2.59) |  |
| Yes | 1260 (24.7) | 3.83 (2.97) |  |
| **PTSD symptoms** |  |  | < 0.001 |
| No | 3741 (73.3) | 1.93 (2.47) |  |
| Yes | 1362 (26.7) | 4.17 (2.95) |  |
| **Investigation period** |  |  | < 0.001 |
| January 29, 2021 – February 28, 2021 | 958 (18.8) | 3.79 (2.91) |  |
| March 1, 2021 – March 31, 2021 | 3223 (63.2) | 2.89 (2.66) |  |
| April 1, 2021 – April 26, 2021 | 922 (18.1) | 2.61 (2.56) |  |

COVID-19, coronavirus disease 2019; PTSD, posttraumatic stress disorder; SD, standard derivation. ^a^ 1 ¥ = USD$0.14
